# Implementing the World Mental Health Survey Initiative in Portugal – rationale, design and fieldwork procedures

**DOI:** 10.1186/1752-4458-7-19

**Published:** 2013-07-09

**Authors:** Miguel Xavier, Helena Baptista, Jorge M Mendes, Pedro Magalhães, José M Caldas-de-Almeida

**Affiliations:** 1 Department of Mental Health, Nova Medical School, Nova University of Lisbon, Lisbon, Portugal; 2 ISEGI-NOVA, Nova University of Lisbon, Lisbon, Portugal; 3 Social Sciences Institute, University of Lisbon, Lisbon, Portugal

## Abstract

**Background:**

The World Mental Health Survey Initiative was designed to evaluate the prevalence, the correlates, the impact and the treatment patterns of mental disorders. This paper describes the rationale and the methodological details regarding the implementation of the survey in Portugal, a country that still lacks representative epidemiological data about psychiatric disorders.

**Methods:**

The World Mental Health Survey is a cross-sectional study with a representative sample of the Portuguese population, aged 18 or older, based on official census information. The WMH-Composite International Diagnostic Interview, adapted to the Portuguese language by a group of bilingual experts, was used to evaluate the mental health status, disorder severity, impairment, use of services and treatment. Interviews were administered face-to-face at respondent’s dwellings, which were selected from a nationally representative multi-stage clustered area probability sample of households. The survey was administered using computer-assisted personal interview methods by trained lay interviewers. Data quality was strictly controlled in order to ensure the reliability and validity of the collected information.

**Results:**

A total of 3,849 people completed the main survey, with 2,060 completing the long interview, with a response rate of 57.3%. Data cleaning was conducted in collaboration with the WMHSI Data Analysis Coordination Centre at the Department of Health Care Policy, Harvard Medical School. Collected information will provide lifetime and 12-month mental disorders diagnoses, according to the International Classification of Diseases and to the Diagnostic and Statistical Manual of Mental Disorders.

**Conclusions:**

The findings of this study could have a major influence in mental health care policy planning efforts over the next years, specially in a country that still has a significant level of unmet needs regarding mental health services organization, delivery of care and epidemiological research.

## Background

### Introduction

Recent epidemiological research shows that psychiatric disorders and mental health-related problems have become the main cause of disability, and one of the main causes of morbidity and premature death throughout the world [1].

Mental disorders are responsible for more than 12% of the global burden of disease in the world as a whole – a figure that rises to 23% in developed countries. Five of the 10 main causes of long-term disability and dependency are neuropsychiatric conditions: unipolar depression (11.8%), alcohol-use disorders (3.3%), schizophrenia (2.8%), bipolar disorders (2.4%) and dementia (1.6%) [2]. In Europe, mental health problems account for nearly 26.6% of the total burden of ill health, while suicide is one of the top ten leading causes of premature death [3]. Estimates from the European Brain Council indicate that 27.4% of the EU population aged 18 to 65 suffer from one type or another of mental health problem during each one-year period [4], a number that has been recently updated to 38,2% after the inclusion of data from a broader childhood and adolescence assessment, as well as from new EU member states [5].

While there are people who have a diagnosable disorder, many others have mental health problems that can be considered “subliminal”, meaning that they do not meet the diagnostic criteria for psychiatric disorders, but are also in distress, and should therefore benefit from intervention.

Furthermore, people with mental health problems are more likely to have physical health problems, leading to a significant impact on family life, social networking, job performance, and employment, as well as to suffer from stigma, discrimination, and social exclusion. In fact, there is reliable evidence that in several places basic human rights may be denied to people with mental health problems [6]. Besides the burden of disease associated with psychiatric conditions, economic impact should also be taken into account: for instance, in the United Kingdom alone, the cost to the economy (direct health costs, welfare benefits, lost productivity at work) was estimated at over £77 billion every year [7].

In the last two decades, psychiatric epidemiological studies provided a relevant contribution to unveil the dimension, determinants, social impact and treatment gap of the psychiatric disorders.

Thanks to the refinement of survey methodology and questionnaire development, it has been possible to carry out studies based on fully-structured interviews of large samples of the general population, administered by trained lay-interviewers, such as the Epidemiological Catchment Area Study [8] and the National Comorbidity Survey [9], which showed rates of prevalence of psychiatric disorders close to 30% in the year prior to the interview.

Ten years ago, the World Health Organization (WHO) and Harvard University jointly decided to promote a worldwide initiative of population-based surveys using the same methodologies – the World Mental Health Survey Initiative (WMHSI) [10] – aiming to improve the knowledge on the natural history, magnitude and impact of mental illnesses. Conducted in more than 30 countries worldwide (in the Americas, Africa, Europe, Western Pacific and South-East Asia), with a total sample size that can exceed 154.000 people, this project has provided so far, through more than 500 published papers, a huge amount of crucial epidemiological information regarding the planning and implementation of mental health policies (for further details please refer to the project’s website, available at: http://www.hcp.med.harvard.edu/wmh).

In 2007, after the launching of a new national mental health plan, it was decided to carry out a national survey in Portugal, in order to overcome the scarcity of representative data about the prevalence of psychiatric disorders and mental health problems in the country. Grounded on the data from the European Brain Council Report entitled “Costs of Disorders of the Brain in Europe”, it has been indirectly estimated that 1,557,054 (16.07% of the adult population - 18 to 65 years) have a mental disorder in Portugal. According to this projection, 5.09% of the Portuguese adult population suffer from affective disorders (including dysthymia), 9.46% from anxiety disorders, and 0.52% from psychotic disorders [4]. Beyond this projection, general psychological morbidity data from the Eurobarometer survey has suggested that the prevalence of mental health problems in Portugal could be higher than in other European countries of similar characteristics, while the most vulnerable groups (women, the poor, the aged) seemed to exhibit also a higher risk of psychiatric caseness than in the rest of Europe [11]. Several other studies, although conducted with non-representative samples, seem to point in the same direction [12-15].

Despite the available data suggesting the existence of significant levels of psychiatric morbidity and unmet needs for care throughout the Country, there are still no sound published figures about the prevalence of psychiatric disorders in Portugal.

To collect comprehensive and representative epidemiological data, a survey following the methodological framework of the WMHSI was carried out in Portugal, between 2008 and 2009. This study, overseen by the WHO and Harvard University, was coordinated by the Nova Medical School (Department of Mental Health, NOVA University of Lisbon). This is the first rigorous general population survey carried out with a nationally representative sample of the Portuguese population, aiming to evaluate the prevalence, the correlates, the impact and the treatment patterns of mental disorders. This paper presents an overview of the methodology implemented in Portugal, covering the main features of the study (design, fieldwork organization, sampling and weighting procedures).

### Country socio-demographic profile at a glance

Portugal is composed of a mainland territory at the western tip of the Iberian Peninsula, and the Azores and Madeira archipelagos in the North Atlantic. The whole territory has a population of 10,5 million people, a density of 114 people/km^2^ and an annual population growth of 0% (fertility rate: 1,4). With an average life birth expectancy of 79, 8 years, people over 65 already represent 18.2% of total inhabitants (compared to 13.8% in 1991) and have thus overtaken the 0–14 year age group (15.1%). Most relevant economic indicators are: GDP per capita, 25,352 USD; economic growth, -1.6%; public expenditure on health, 7.1% of GDP; public social expenditure, 25.2% of GDP; unemployment rate, 17.8% of total labour force [16]. Despite the improvement in socio-economic conditions that has occurred over the last three decades, there is a large degree of inequality in net annual incomes: the most significant incidences of poverty are to be found among people who are aged over 65 and live alone.

## Methods

### Design and general framework

Following the original methodology designed by the WMHSI [10], the Portuguese mental health survey is a cross-sectional study based on stratified multistage clustered area probability household sample. It was carried out at the households of a Portuguese nationally representative sample of respondents, between October 2008 and December 2009. The survey was administered by trained lay-interviewers on a face-to-face setting, using the computer-assisted personal interview (CAPI) methodology. The use of CAPI rather than the paper-and-pencil (PAPI) version was a decision from the WMHSI Data Collection Coordination Centre (Harvard University), in order to avoid problems related with data input costs, increased length of interview and dropping-out, as well as to facilitate quality control. Given the complexity of the sampling procedures and the fieldwork, a highly specialized survey unit (Center for Public Opinion Studies and Polls, CESOP) belonging to the Portuguese Catholic University, was selected to implement the protocol throughout the Country, under the scientific coordination of the Nova Medical School. The project was submitted to and approved by the Ethics Committee of the Nova Medical School in January 2008.

### Target population

The National Census 2001, published by Statistics Portugal [17], was used to estimate the target population of mainland Portugal in 2008. The target population for the survey was defined as the usually resident, non-institutionalized Portuguese-speaking population of Continental Portugal aged 18 or above, residing in permanent private dwellings.

This definition excluded: a) People living in non-private dwellings, b) Residents of rest homes, hospitals and psychiatric institutions, c) Military personnel not residing in a private dwelling, d) Prison inmates, e) non-Portuguese speakers and f) Other people unable to answer the questionnaire.

Recent data from the National Census 2011 [18] confirms that the population that meet the criteria a) to d) represents 1.3% of the total population aged 20 years or more; the population that meet the criteria e) is unknown, but Census 2011 estimates it at around the 1% level. Anyway, two limitations have remained: 1. the above percentage of 2.3% (1.3% + 1.0%) was measured in 2011 and not in 2008, although it is not expected that major changes had occurred between 2008 and 2011 and 2. the size of the population belonging to criteria f) is not known.

### Sampling

This is a stratified four-stage clustered area probability design, using the “locality” as the primary sampling unit (PSU). Unlike what happens in several countries, which have reliable lists of residents available for survey research – such as Sweden, for example – or where there are reliable lists of households/addresses – such as the UK, for example – in Portugal no such lists are available. Therefore, a multistage design needs to be applied, in which the selection of localities forms the first stage. “Localities” are territorial delimitations defined in the context of census operations by the Portuguese national statistics office – Statistics Portugal – consisting in population clusters with 10 or more residential dwellings and to which a distinct place name is attached. For each locality, the number of households and persons 15 years and older is known on the basis of census data. According to the Census 2001, 27,960 localities existed in Portugal mainland, with 7,719,986 inhabitants aged 18 or more. At stage 1, 262 primary sampling units (PSU) were randomly selected with probability proportional to size (PPS), as shown in Table 1. Selection was stratified by region (North, Centre, Lisbon, Alentejo, and Algarve) and size of locality (1- ≤ 2,000 inhabit.; 2–2,000-9,999 inhabit.; 3–10,000–19,999 inhabit.; 4 – 20,000 – 99,999 inhabit.; 5– ≥100,000 + inhabit.). The number of non-empty strata created was of 23 (there were no localities with 100,000 inhabitants or more in the regions of Alentejo and Algarve).

**Table 1:** Number of localities randomly selected (stratified by region and locality size)

**Region**	**Size of number of inhabitants in the localities (PSU)**				
	**Less than 2,000**	**2,000 – 9,999**	**10,000 – 19,999**	**20,000 – 99,999**	**100,000 or more**
**North**	53	15	9	20	2
**Centre**	48	9	5	6	1
**Lisbon**	12	18	11	17	2
**Alentejo**	10	8	2	2	
**Algarve**	6	2	2	2	
**Total**	262				

Out of the 262 PSU’s selected, 52 PSU’s entered the sample with certainty. The certainty selection included all localities with population larger than 20,000 inhabitants. Those 52 PSU’s are referred to as ‘self-representing’ PSU’s because they were not selected randomly to represent other localities, but they are so large that they represent themselves. The remaining ones are ‘non-self representative’ because they were selected to be representative of smaller areas of the country. In the end, the PSU’s with interviews were distributed as shown in Table 2.

**Table 2:** Number of localities (stratified by region and locality size) where interviews were conducted

**Region**	**Size of number of inhabitants in the localities (PSU)**				
	**Less than 2,000**	**2,000 – 9,999**	**10,000 – 19,999**	**20,000 – 99,999**	**100,000 or more**
**North**	54	9	16	19	2
**Centre**	46	4	9	6	1
**Lisbon**	12	11	17	15	2
**Alentejo**	11	1	8	2	
**Algarve**	6	2	1	2	
**Total**	256				

At stage 2, there was a selection of random-route starting points. By means of aerial maps, coordinates were randomly selected. Initially, for PSU’s with less than 100,000 inhabitants, a total of 4–6 routes starting points were selected. For the ‘self-representing’ PSU’s above 100,000 inhabitants a number between 12 and 43 starting points were selected.

At stage 3, the initial selection of households was conducted. Using the already mentioned 2001 Census information on the number of households in each locality, households were selected by applying intervals proportional to size of locality and divided by number of random-route points to be selected in each locality. This design creates a sample in which the probability of any individual Housing Unit (HU) being selected to participate in the survey is equal to every HU in Portugal mainland. A total of 10,067 addresses were selected.

At stage 4, information regarding the number of people aged 18 years old or more living in the household was collected. The interviewers registered each selected address and, if someone was present, registered gender and birth date of qualified members of the population in each household, leaving information about survey and collecting phone numbers. Following analysis of interviewer records and validation of choice of household and/or respondent by CESOP central coordinators, based on the last birthday method (in which an interview is attempted with the adult in the household who had the most recent birthday), 8,253 respondents were selected, under the expectation of a 50% cooperation rate.

### Tools and measures

The interview tool is the WMH-CIDI, a new expanded version of the WHO-Composite International Diagnoses Interview (CIDI), developed by the WMHSI and the National Institute for Mental Health [19]. The original CIDI is a fully structured questionnaire on the presence, persistence and intensity of clusters of psychiatric symptoms and provides, by means of computerized algorithms, lifetime and 12-month mental disorders diagnoses according to the International Classification of Diseases (ICD-10) [20] and accordingly with the DSM-IV [21]. The WMH-CIDI was developed in order to overcome detected shortcomings of the original version, and was extended to include accurate questions about disorder severity, impairment, and treatment. DSM-IV disorders are ranked as serious if include at least one of the following: criteria for bipolar I disorder or substance dependence with a physiological dependence syndrome; suicide attempt in conjunction with any other WMH-CIDI/DSM-IV disorder; at least two areas of role functioning with severe role impairment due to a mental disorder in the disorder-specific Sheehan Disability Scales; or reporting overall functional impairment at a level consistent with a Global Assessment of Functioning of 50 or less in conjunction with any other WMH-CIDI/DSM-IV disorder [10].

Kessler et al. [22] and Haro et al. [23] provided evidence that diagnoses of anxiety, mood, and substance disorders based on CIDI 3.0 have generally good concordance with diagnoses based on blinded clinical reappraisal interviews.

The adaptation of the original WMH-CIDI to Portuguese was conducted by a committee from the Nova Medical School, including 10 bilingual experts with clinical experience, coordinated by two of the authors (JMCA and MX), in close contact with the WMHSI Data Collection Coordination Centre. The process was driven according to 5 specific dimensions: semantic equivalence (likeness of meaning of each item), content equivalence (item cultural relevance), technical equivalence (use of the same measuring techniques), conceptual equivalence (relationship of the theoretical constructs against criteria known to be related) and criterion equivalence (similarity of the results of the measure in the two cultures). To achieve the Portuguese final version, a step-by step procedure was used as detailed below.

1st step: The process started with a general review of all sections, aiming at the identification of words, phrases and idiomatic expressions not commonly used in Portugal, and listing of suggested language equivalents.

2nd step: A specialized review of each section was conducted by two members of the committee, according to their specific areas of expertise. The reviewers were asked to continue the identification of words, phrases and idiomatic expressions not commonly used in Portugal, and to make recommendations for modifications to the Portuguese version, aiming for equivalence (semantic, content, conceptual, and technical) with the original English version.

3rd step: The instrument was then reviewed item by item by a group (JCA+ MX + the persons responsible for the second review) focusing on the modifications made to fit the vernacular use in Portugal and working for consensus on items that were thought to be problematic.

4th step: All sections and items were crosschecked by the same group for consistency in word use throughout the instrument.

5th step: A new version was created with all of the agreed upon cultural adaptations.

6th step: The entire newly adapted instrument was administered to 71 potential respondents of the target population to test for respondents’ reactions and understanding of particular questions that people do not seem to understand or seek clarifications on.

7th step: Last changes and confirmation of the final version by JMCA+MX, after results from the pilot test.

Disorders considered in the Portuguese version of the WMH-CIDI include anxiety disorders (agoraphobia, generalized anxiety disorder, obsessive-compulsive disorder, panic disorder, posttraumatic stress disorder, social phobia, specific phobia), mood disorders (bipolar I and II disorders, dysthymia, major depressive disorder), disorders that share a feature of problems with impulse control (bulimia, intermittent explosive disorder - for all respondents - and adult persistence of childhood/adolescent disorders-attention deficit/hyperactivity disorder, conduct disorder, and oppositional-defiant disorder - only for respondents in the 18 to 44-year age range), and substance disorders (alcohol abuse and dependence).

In addition, the instrument includes modules intended to assess, amongst others, various areas of life, including marriage, work, financial issues and education. Other modules included are the functioning and physical disorders (stigma, discrimination, number of days unable to work, diseases severity, childhood adversities and suicide) and the treatment (psychiatric treatment, other mental health treatment, any professional treatment, any health treatment, and reasons for no treatment).

Once prepared, the content of the Portuguese version was transferred to a computer-assisted personal interview application (CAPI) using the Blaise software [24], a task that involved reprogramming the codes received from the WMHSI Data Collection Coordination Centre. Special attention was given to the skips and jumps within and between sections, in order to fully respect the original CAPI algorithms.

A pilot study was conducted in May 2008, to evaluate the general adequacy of the instrument and to obtain feedback from respondents regarding the interview. Specific aims were to test the selection of respondent procedures, to identify difficulties in the understanding of items and to detect any problem related with the software, particularly if associated to skips, jumps and spelling.

The instrument was pre-tested in 71 respondents living in the district of Lisbon. The distribution of the people on the sample was based in the distribution of the Portuguese population presented in the Census 2001, thus representing all adult age groups, males and females, from different socio-economic strata. Interviews were conducted by 10 previously trained lay-interviewers, appointed by the CESOP survey centre to carry on the supervision of the field work.

The data from the pilot study was sent to the WMHSI Data Collection Coordination Centre on May 2008. During the data cleaning some skip errors were found both within and between sections, which led to several amendments introduced in the final version of the Portuguese WMH-CIDI CAPI.

Besides minor problems related with some questions considered as being confuse, repetitive and too long, the main problem found was that the complete interview could take up to 180 minutes. Internal sub sampling was used to reduce respondent burden by dividing the questionnaire into two parts. During the field work, all respondents completed Part I, which included screening questions and assessed core mental disorders. All respondents that met the criteria for any DSM-IV disorder were then administrated Part II, the diagnostic, additional disorders and correlates modules. Beyond that, Part II was also administrated to a probability sample of 25% randomly selected by the Blaise Code of those who did not meet criteria for any disorder. Therefore, to out of a total of 3,849 interviews, 2,060 were administrated the ‘long interview’ (Part I + Part II) and the remaining ones were administrated the ‘short interview’ (Part I).

### Fieldwork organization and procedures

#### Training

As noted before, lay interviewers carried out the fieldwork. To supervise their activities, a group of 18 Supervisors and Coordinators was trained during a full week (30 hours), in April 2008. Training schedule for future supervisors and coordinators included lectures on the whole CIDI, followed by group-work focused on the sections previously presented. Lectures were also given about fieldwork procedures, interviewing technique and on how to use the CAPI program. In the last day of the training the interviewers were expected to undertake a first complete mock interview under the supervision of a trainer from Nova Medical School. Following the training week, the interviewers had to complete 3 additional interviews before being accepted by the CESOP centre.

Interviewers were also trained during full-time periods of 5 days. Training was conducted in groups of around 15 people, and embodied two parts: general interviewing techniques (GIT) and description of the WMHSI protocol (study-specific training). Regarding GIT training, interviewers were firstly introduced to the basic components of standardized questionnaire administration, covering topics such as question reading, appropriate techniques for probing for more information, seeking clarification, providing feedback, and accurate data recording/data entry. Special attention was given to reluctance handling techniques, standardized interviewing techniques, interviewer evaluation procedures and sample management. All trainees were required to demonstrate competence with GIT concepts and procedures before moving on to study-specific training. Learning assessment methods included direct observation of scripted interviews, role-playing exercises and a variety of written assignments and oral tests.

Regarding study-specific training, a mix of lecture and round-robin practice sessions were presented, addressing the following issues: general background of the project, administration of the survey, introduction and elements of informed consent, eligibility and respondent selection procedures, interviewing on sensitive topics, and how to meet production goals.

Tailored “after-hours” special sessions were held to address areas where trainees need additional assistance and practice, such as in applying the respondent selection steps and using the CAPI. Trainees were required to pass a certification test before being approved as interviewers: to those interviewers who did not pass, additional re-training was provided as a last opportunity to obtain certification.

#### Procedures

After sample selection, each selected household received a brochure asking for participation in the study, presenting the objectives, the work team and the free phone number to contact the supervisors’ team. An introduction letter, signed by the Principal Investigator, was sent at the same time. At the first visit, and after confirming availability, household was centrally confirmed and a phone contact was undertaken to schedule interview. If availability was not confirmed either new visits were conducted or a refusal was registered (see below, for call procedures detailing fieldwork guidelines regarding household visiting, interview scheduling and substitutions).

### Call procedures (fieldwork guidelines regarding household visiting, interview scheduling and substitutions)

• If the household report available after first visit and selection of household is centrally confirmed, contact by phone is made to schedule interview;

• If household report not available after first visit, subsequent visit is made to fill household report; number of visits before household deemed “unknown if occupied”: 9

• Number of established contacts with household with failure to contact selected respondent before deemed “non-contact”: 5 phone contacts, and 4 household visits before deemed “non-contact”.

• Number of scheduled interviews with failure to attend by respondent before deemed “refusal”: 2

• No substitutions: all respondents are extracted from initially selected households.

Table 3 shows the final sample disposition. From the total 10,067 households selected, 3,849 participated in the survey, representing 57.3% of total contacted (6,714). The response rate achieved was close of that achieved in Belgium, France, Germany, and Netherlands, but lower than that achieved on others countries, like the USA [25]. No substitutions were allowed, meaning that all respondents were extracted from the initially selected household. Informed consent was asked and obtained in every occasion, by means of a signed form, previously accepted by the Nova Medical School Ethics Committee.

**Table 3:** Sample distribution

**Sample distribution**	**%**	**(n)**	**%**
**Interview**	38.2%	3,849	57.3%
**Refusal**	28.5%	2,865	42.7%
**Sub-total**		6,714	
**No contact**	14.0%	1,408	
**Circumstantial (outside the sample definition)**	17.0%	1,707	
**Others**	2.3%	238	
**Total**		10,067	

The interviewers were paid with a fix amount to accomplish the total number of interviews. Productivity premiums were also paid monthly based on goals achievement and quality of work delivered. On the first 1,500 interviews the respondents were given a 15€ gift-card as a token of appreciation for participating in the survey. Trying to increase participation, the remaining interviews were given a 30€ gift-card.

Supervisors spent at least 2 hours a week with all interviewers and every two weeks had a meeting with all interviewers to assess progress, plan the work for the coming weeks and distribute materials.

The Collection Coordination Centre, from the University of Michigan’s Survey Research Centre at the Institute of Social Research, guaranteed consistency in survey implementation by developing and training collaborators. As an example of that work, internal consistency checks of survey responses were carried centrally and provided to supervisors.

#### Data management and quality control

Once surveys were completed, data was sent to the WMH Data Analysis Coordination Centre at the Department of Health Care Policy, Harvard Medical School, for data cleaning. The first data file of the study was sent in July 2008, containing 711 completed interviews. The cleaning process revealed minor problems concerning a small number of CAPI outputs, namely due to incorrect skips between questions/sections of the CIDI, that were easily corrected.

Once cleaning was completed, centralized coding and analysis were carried out. Cleaned and coded data sets and the results of preliminary analysis were sent back to the Portuguese team.

A comprehensive system of quality assurance was set from the starting of the fieldwork, including several simultaneous mechanisms. Appointments were monitored by random call-backs to households (10% of total) using part of the stem questions in the ‘Screening’ section, as well as other questions created by the management team (ex. “*Did you receive the incentive? How much was it?*”). During these random call-backs, supervisors evaluated i. study eligibility i.e. if the appropriate respondent was correctly interviewed (instead of another household resident), ii. response to key questionnaire items , iii. date, time and total length of the interview (to cross-check with the duration displayed by the software program), iv. respondent’s feedback on interviewer’s professionalism and v. compliance with the interviewing rules and guidelines set forth in the training. The WMHSI Coordinating Centre provided a software that use the clocks in the laptops to time data entry as a way to detect possible interviewer cheating, also allowing the rapid analysis of computerized CAPI interviews to detect missing values and other signs of low interview quality. A deliberate violation of interview guidelines was found in one occasion, leading to immediate exclusion of the interviewer. Besides the indirect evaluation, fieldwork supervisors from the CESOP directly observed up to 10% of all interviewers’ work, selected randomly from the case register. Monitoring was more frequent earlier in the study, but to ensure reliability supervisors continued to monitor even the experienced interviewers till the end of the study, in addition to the less experienced interviewers.

On a daily basis, supervisors conducted questionnaire review of finished interviews, checking for general congruence, missing data, jumping inconsistencies or other problems. Detailed feedback was given to interviewers no later than 3 days, with a special emphasis on detection and prevention of falsified information, conformity with the interviewing conventions and guidelines, performance of non-interview tasks and identification of interviewer-questionnaire interface problems.

Finally, meetings attended by all the staff (interviewers, supervisors) and coordinated by one of the authors (JMCA or MX) were held on a monthly basis, aiming to address general problems found in the fieldwork and particularly to ensure the maintenance of strong levels of cohesion and motivation within the group.

### Weighting

When conducting a survey, having a representative sample of the population is of paramount importance, but despite the best efforts some characteristics (such as age, education, race, gender, etc.) of the sample might be accidentally oversampled or under-sampled. Correction can be accomplished via a post-stratification.

On the present data, two different weightings were considered. WT1 will be used when the total sample (n=3,849) is considered, while WT2 will be used just for the respondents answering the long interview (n=2,060).

WT1 weighting was calculated based on two different weights. Firstly, the number of eligible respondents in each household was computed, allowing to the calculation of the within-household weight (weight 1 of WT1). The within-household probability of selection weight adjusts for the fact that the probability of selection of respondents within the HU varies inversely with the number of people in the HU. This is true because, as noted earlier, only one respondent was selected for interview in each HU. Three variables in the household listing were included at the individual-level records: respondent age and gender and number of eligible residents in the HU. If the number of eligible respondents in the household was greater than 5, then 5 was used. This was the weight 1 of the WT1.

To compute the second weight (weight 2 of WT1), and adjust for variation between the joint distribution of age-gender in this weighted sample compared to the INE published data, a post-stratification by those two socio-demographic variables for each of the 5 regions of Portugal was done.

WT1 is equal to weight 1 × weight 2. The sum of the WT1 is now the population size of the 5 regions in Portugal mainland, aged 18 or more, as it was published by Statistics Portugal, as the annual Portuguese resident population for 2008, in September 15, 2009. This weight was then normalized to the sample in order to obtain the final WT1. After that, the upper and lower 3% of cases, in the final WT1, were trimmed by assigning them the average value of the total weight and therefore obtaining the “weight trim”. After this, the “weight trim” becomes the final WT1.

Comparison of the unweighted distributions of the sample with the Statistics Portugal published data distributions provides information justifying the weighting above described. As Table 4 shows, the sample overrepresented women and people with 35–64 years of age – these distortions were corrected with the WT1 weight.

**Table 4:** Unweighted sample composition and National Institute for Statistics (INE) published data

**Gender/Age**	**Unweighted**	**INE**
	**%**	
**Gender**		
**Male**	42.40	48.30
**Female**	57.60	51.70
**Age**		
**18-34**	27.18	29.44
**35-49**	30.27	28.27
**50-64**	25.51	23.63
**+65**	17.04	18.66

WT2 weighting was also calculated based on two different weights. Firstly, a group was assign to each respondent, ‘core disorders’ or ‘no core disorders’. 572 respondents without core disorder got the long interview – in fact, they represent a total of 2,342 respondents that did not have a core disorder (572 without core disorder that got the long interview + 1,770 without core disorders that did not got the long interview ). Therefore, weight1 of WT2 is ‘1’ for the interviews that have a core disorder and ‘572/2.342’ for the ones who do not have a core disorder. After multiplying those values by WT1, the final weight 1 of WT2 was obtained.

The weight 2 of WT2 was calculated exactly the same way as weight 2 of WT1 (based on age and gender, on regional level). WT2 was firstly calculated multiplying weight 1 × weight 2, followed by normalization of the interview sample of 2.060 (long-interviews).

Depending if a disorder is included in the long or short interview, WT1 or WT2 will be used accordingly in order to calculate the prevalence.

## Discussion

The implementation of the WMH survey in Portugal was a difficult process, but also a very important challenge for the teams involved in the project. Generally, the type of problems and limitations faced during the implementation were rather similar to the ones already described by other countries belonging to the WMHSI Initiative [26]. Firstly, this is a very demanding, time-consuming and costly project, with methodological requirements highly specific and quite demanding. Cost-efectiveness has been pointed, in fact, as one of the major problems of the WMH surveys, with some authors arguing that these cross-sectional studies might not be cost-effective [27], once relying in potentially biased retrospective reports. Secondly, it is plausible that people with serious mental disorders could be more prone to refuse being interviewed (i.e., systematic nonresponse), which could lead to bias regarding the estimation of disorder prevalence. The same applies to non-reporting, an issue that is often relevant in very lengthy interviews, in which the respondents may get tired or bored.

Looking from a more positive perspective, the relatively small size of the country and the quality of the data provided by Statistics Portugal were undoubtedly factors that facilitated the fieldwork. As well, group cohesion and motivation ensured by the CESOP were crucial ingredients to the success of the study, avoiding a high turn-over of interviewers and thus decreasing the need for further CIDI training during the project.

The coordination team was in close contact with Harvard University during the whole study, which allowed an excellent scientific support in the data management process, namely on data cleaning. Two of authors (JMCA, MX) have participated in the Annual WMHS Meetings since 2008, presenting updated data on the implementation of the study in Portugal.

Results, prevalence rates and correlates analysis will be published soon after the publication of this paper about general methodology. The results of this study may be of great importance to the Portuguese authorities, as it will provide the Government with the first nationally representative data on the magnitude and distribution of the mental health disorders in Portugal. In fact, despite some indubitably positive aspects, due to lack of planning and consistent support in the improvement of mental health services, Portugal is still lagging behind in this field in relation to other European countries. Existing data and analysis of results from research undertaken as part of the National Mental Health Plan Report show that mental health services suffer from serious deficiencies, in terms of accessibility, equity and quality of care. Many local mental health services continue to be limited to hospitalization, outpatient consultations and, sometimes, day hospital, and have no community mental healthcare teams, with integrated case management, crisis intervention and programs involving families. On the other side, the number of people in contact with public services shows that only a small part of those with mental health problems have access to specialized mental health services. Even assuming that only people with severe mental illnesses attend mental health services – which we know is not the case – the number of contacts (17% of the population) is still extremely low in relation to what should be expected.

This study will provide currently unavailable sound data on patterns and correlates of service use and barriers to obtaining available treatment.

## Conclusions

This is the first general population survey of psychiatric morbidity conducted in a nationally representative sample of the Portuguese population. The findings of this study can have a major influence in mental health care policy planning efforts over the next years, specially in a country that still has a significant level of unmet needs regarding mental health services organization, delivery of care and epidemiological research.

## Competing interests

The authors declare that they have no competing interests.

## Authors’ contributions

MX participated in the preparation of the study and the coordination of the project and drafted the manuscript. HB and JMM calculated the weightings, and helped in drafting the manuscript; PM participated in the fieldwork coordination and data management; JMCA was the PI of the WMH survey in Portugal, led the adaptation of the tools and coordinated the whole project. All authors have reviewed critically and approved the text of the manuscript.
